# *In Silico* Analysis of Genetic VapC Profiles from the Toxin-Antitoxin Type II VapBC Modules among Pathogenic, Intermediate, and Non-Pathogenic *Leptospira*

**DOI:** 10.3390/microorganisms7020056

**Published:** 2019-02-20

**Authors:** Alexandre P. Y. Lopes, Bruna O. P. Azevedo, Rebeca C. Emídio, Deborah K. Damiano, Ana L. T. O. Nascimento, Giovana C. Barazzone

**Affiliations:** 1 Laboratório Especial de Desenvolvimento de Vacinas—Centro de Biotecnologia, Instituto Butantan, Avenida Vital Brazil, 1500, 05503-900 São Paulo, Brazil; bruna.azevedo@butantan.gov.br (B.O.P.A.); rebeca.emidio@butantan.gov.br (R.C.E.); dkohndamiano@gmail.com (D.K.D.); ana.nascimento@butantan.gov.br (A.L.T.O.N.); giovana.barazzone@butantan.gov.br (G.C.B.); 2 Programa de Pós-Graduação Interunidades em Biotecnologia, Instituto de Ciências Biomédicas, USP, Avenida Prof. Lineu Prestes, 1730, 05508-900 São Paulo, Brazil

**Keywords:** Toxin-antitoxin, VapBC, VapC, *Leptospira*

## Abstract

Pathogenic *Leptospira* spp. is the etiological agent of leptospirosis. The high diversity among *Leptospira* species provides an array to look for important mediators involved in pathogenesis. Toxin-antitoxin (TA) systems represent an important survival mechanism on stress conditions. *vap*BC modules have been found in nearly one thousand genomes corresponding to about 40% of known TAs. In the present study, we investigated TA profiles of some strains of *Leptospira* using a TA database and compared them through protein alignment of VapC toxin sequences among *Leptospira* spp. genomes. Our analysis identified significant differences in the number of putative *vap*BC modules distributed in pathogenic, saprophytic, and intermediate strains: four in *L. interrogans*, three in *L. borgpetersenii*, eight in *L. biflexa*, and 15 in *L. licerasiae*. The VapC toxins show low identity among amino acid sequences within the species. Some VapC toxins appear to be exclusively conserved in unique species, others appear to be conserved among pathogenic or saprophytic strains, and some appear to be distributed randomly. The data shown here indicate that these modules evolved in a very complex manner, which highlights the strong need to identify and characterize new TAs as well as to understand their regulation networks and the possible roles of TA systems in pathogenic bacteria.

## 1. Introduction

Leptospirosis is caused by pathogenic spirochetes of the genus *Leptospira*. It is a zoonotic disease affecting humans and a wide range of animals worldwide with significant impact. The genus *Leptospira* comprises saprophytic and pathogenic species (family Leptospiraceae, order Spirochaetales) and were named based on their spiral shape. They are mobile and measure 6 to 20 μm in length by 0.1 μm in diameter [1,2]. The most serious manifestation of pathogenic leptospirosis results in a syndrome known as Weil’s disease, which is characterized by a devastating kidney and liver failure [2,3].

Based on 16S rRNA phylogeny, DNA-DNA hybridization, pathogenicity, virulence, and in vitro growth characteristics, the genus *Leptospira* includes at least 21 species arranged in three groups: pathogenic, intermediate pathogenic, and non-pathogenic or saprophytic [4,5], which in turn are divided into over 200 serovars defined by agglutination with homologous antigen [3]. Group I pathogens produce disease in people, mostly severe, caused by bacteria belonging to the evolutionarily-related species *L. interrogans*, *L. kirschneri*, and *L. noguchii*. Group II intermediate pathogens grow better in culture and cause predominantly mild self-resolving illnesses without fatal complications. Group III saprophytic *Leptospira* are free-living environmental microorganisms [4].

Toxin-antitoxin (TA) systems represent an important mechanism of bacteria survival during stress conditions, such as starvation or antibiotic pressure. TA operons are widely distributed among bacteria and are characterized by a pair of genes encoding for a stable toxin and an unstable antitoxin. The antitoxin acts as an antagonistic regulator that prevents the toxin from exerting its toxicity, except when some environmental conditions determine a decrease in antitoxin concentration, exposing the cell to toxic effects, leading to a reversible cessation of growth [6,7]. TA systems have been involved in potentially harmful aspects of an infection, such as antimicrobial resistance, persistence, and biofilm formation [8]. In some pathogens, including *L. interrogans* [9], *Mycobacterium tuberculosis* [10,11], *Escherichia coli* [12], *Haemophilus influenzae* [13], and *Salmonella enterica* [14], they participate in the bacterial physiology during the infection.

Experimental data of toxin-antitoxin and in silico analysis on prokaryotic chromosomes have shown the widespread presence of TA modules among bacteria with few exceptions, like the spirochetes *Borrelia burgdorferi*, *Treponema pallidum*, and other obligated host associated bacteria [15,16,17,18]. TAs are classified into types based on the nature—nucleic acid or protein—of their toxin and antitoxin and on the kind of interaction between them. To date, six types of TA systems have been described [19,20], with the type II system being the most abundant. Type II TA systems are composed of an inhibitory proteic antitoxin that interacts with the toxic protein [21].

Type II TA modules are grouped into different families according to the toxin structure and protein sequence similarity [6,22]. VapBC is the main TA type II family, with about 1900 VapBC modules identified in 960 genomes, corresponding to 30–40% of the known TA modules (URL:http://bioinfo-mml.sjtu.edu.cn/TADB/) [23]. They are classified based on the presence of a PIN domain (PilT N-terminal) VapC, which is presumed to confer ribonuclease activity to the toxin. Like VapCs, toxins of the RelBE, MazEF, and HicAB families have been described as endoribonucleases, also called interferase RNAs [20,24], which hydrolyze different and specific RNA targets. The RelE toxin cleaves mRNA in the ribosomal A site with high codon specificity [25]. The HicA toxins also cleave mRNA, but independently of the ribosome [26]. Toxins of MazF family have been shown to cleave mRNA [24], rRNA [27], and also tRNA [28]. Most of the few characterized VapC toxins have been shown to exert their activities on the initiator tRNA in a very specific manner. Up until now, the initiator tRNA (tRNAfMet) has been the specific biological target found in the largest number of bacterial species: *Leptospira interrogans* [29], *Salmonella enterica* [30], *Shigella flexneri* [30], and *Haemophilus influenzae* [31]. Other specific tRNA, such as tRNACys-GCA, tRNALeu-CAG, tRNASer-TGA, CGA, and tRNATrp-CCA, have been identified as substrates of VapCs of *Mycobacterium tuberculosis* [32]. Additionally, two VapCs of *M. tuberculosis* cleave 23S rRNA at the sarcin-ricin loop (SRL) [32,33].

The substantial progress of genomic and proteomic studies has allowed for high-throughput studies of leptospiral proteins aimed mainly at the identification of potential antigens for vaccine and diagnostic development [34]. Most of the studies on the genome of leptospiral species have focused mainly on the search for surface-exposed antigens or important proteins to vital metabolic routes [35,36,37,38]. More recently, TA modules have achieved more prominence [39,40]. The high diversity among *Leptospira* species makes their studies very complex and challenging, providing a rich ground to look for specific mediators with importance for bacterial virulence and pathogenesis. In this work, we have investigated the diversity among toxin-antitoxin type II systems among *Leptospira* species, with a focus on the toxin of the VapBC family. We searched for and compared the putative TA operons of the whole sequenced genomes of pathogenic *L. interrogans* and *L. borgpetersenii*, intermediate-pathogenic *L. licerasiae*, and saprophytic *L. biflexa* strains within 20 *Leptospira* ssp., classified according to the pathogenicity phylogenetic tree [5]. This extensive analysis aimed to study the conservation of these TA modules and to evaluate a possible correlation of their presence in the three phenotypes of pathogenicity.

## 2. Materials and Methods

### 2.1. Analysis of Type II TA Modules

The analyses of bacterial type II toxin-antitoxin loci were performed using TADB 2.0 database (http://bioinfo-mml.sjtu.edu.cn/TADB2/) [23]. To explore the whole set of putative TA operons of each *Leptospira* we have used the tool TAfinder (http://202.120.12.133/TAfinder/TAfinder.php) from TADB by entering the Refseq Accession Number of the following bacteria: *L. interrogans* serovar Copenhageni strain Fiocruz L1-130 (Refseq NC_005823), *L. interrogans* serovar Lai strain 56601 (Refseq NC_004342), *L. borgpetersenii* serovar Hardjo-bovis strain JB197 (Refseq NC_008510) and strain L550 (Refseq NC_008508), *L. biflexa* serovar Patoc strain Patoc 1 (Ames) (Refseq NC_010842) and strain Patoc 1 (Paris) (Refseq NC_010602). The set of TA modules of *L. licerasiae* serovar Varillal strain VAR010 was obtained from the manuscript of Ricaldi et al. [39].

### 2.2. Evaluation of the Presence of vapBC among Leptospira spp.

To evaluate the presence of putative *vapBC* operons in the 20 *Leptospira* spp. comprised in this study, we used the program Basic Local Alignment Search Tool (BLAST) (https://blast.ncbi.nlm.nih.gov/Blast.cgi), specifically the protein-protein BLAST tool (BLASTp), to compare the protein sequences of the putative VapC toxins. The VapC query sequences were obtained directly from the TADB database or through locus identification and numbered according to the order of appearance on each genome (base pair numbering) of *L. interrogans* serovar Copenhageni strain Fiocruz L1-130 (LIC), *L. borgpetersenii* serovar Hardjo-bovis strain JBL97 (LBJ), *L. licerasiae* serovar Varillal strain VAR 010 (LEP1GSC185), and *L. biflexa* serovar Patoc strain Patoc 1 (Ames) (LBF). The subject taxonomic ID (taxid) of the *Leptospira* used to browse homologous proteins were: *Leptospira interrogans* serovar Copenhageni str. Fiocruz L1-130 (taxid:267671), *L. interrogans* serovar Lai str. Lai (taxid:1049911), *L. borgpetersenii* serovar Hardjo-bovis str. JB197 (taxid:355277), *L. borgpetersenii* serovar Hardjo-bovis str. L550 (taxid:355276), *L. biflexa* serovar Patoc strain ‘Patoc 1 (Paris)’ (taxid:456481), *L. biflexa* serovar Patoc strain ‘Patoc 1 (Ames)’ (taxid:355278), *Leptospira* (taxid:171), *L. interrogans* (taxid:173), *L. kirschneri* (taxid:29507), *L. noguchii* (taxid:28182), *L. borgpetersenii* (taxid:174), *L. weilii* (taxid:28184), *L. santarosai* (taxid:28183), *L. alexanderi* (taxid:100053), *L. alstoni* (taxid:28452), *L. kmetyi* (taxid:408139), *L. wolffii* (taxid:409998), *L. licerasiae* (taxid:447106), *L. inadai* (taxid:29506), *L. fainei* (taxid:48782), *L. broomii* (taxid:301541), *L. wolbachii* (taxid:29511), *L. meyeri* (taxid:29508), *L. biflexa* (taxid:172), *L. vanthielii* (taxid:293085), *L. terpstrae* (taxid:293075), and *L. yanagawae* (taxid:293069).

#### *“Conservation Value”* Index

In order to easily visualize the conservation between two VapCs sequences, we established the “*Conservation value*” (*C-value*) formed from values provided by BLASTp analyses, expressed as a frequency between 0 and 1. For each query protein, the *C-value* was calculated as follows:

(1) $$C\;value=\frac{\left(i+p\right)}{2}\;x\;c$$

+ where “*i*” is the amino acid identity, “*p*” refers to a positive or similar amino acid, and “*c*” is the amino acid coverage sequence, given by the ratio between the length of the highest scoring matching sequence and the query length. Arbitrarily, we considered sequences with the result of “*p*” and “*c*” > 0.5 as having a considerable degree of “*C-value*”.

### 2.3. Protein Alignment

Alignment between two amino acid sequences and identity (%) were performed using the tool Global Align from BLAST, and multiple sequences alignment was computed using COBALT (Constraint-based Multiple Alignment Tool) (https://www.ncbi.nlm.nih.gov/tools/cobalt/re_cobalt.cgi), which considers the proteic conserved domain and sequence similarity information.

## 3. Results

We have been working on the genomics and proteomics of *L. interrogans* serovar Copenhageni strain Fiocruz L1-130 [5,35,41]. Therefore, this strain was chosen as the basis for the comparative studies presented here. Furthermore, we have studied the TA profile of the sequenced genomes of the pathogenic species *L. interrogans* serovar Lai [37] and two strains of *L. borgpetersenii* [42], as well as two strains of the saprophytic *L. biflexa* [43], available in the TADB database. In the case of the intermediate pathogenic *L. licerasiae*, a detailed TA profile was described in its genome analysis manuscript [39]. Briefly, 28 TA modules were identified, which code for 15 VapBC, one HigAB, one ChpIK, one MazEF, five ArsR/Aha1, one HTH/Aha1, one XRE/COG2856, one RHH/UNK, one RHH/COG2929, and one RHH/DUF497. We have focused this work on the study of the VapBC family because it is the most abundant type II TA family, comprising about 40% of the identified TA modules in bacterial genomes, allowing us to discuss the variability of TA systems in *Leptospira*. Moreover, data from the International *Leptospira* Genome Project [5], made it possible to relate TAs of the genome sequences of 20 *Leptospira* species in order to build up several comparative tables.

### 3.1. Analysis and Comparison of TA Profiles within Leptospira Species

#### 3.1.1. *L. interrogans* Serovar Copenhageni Fiocruz L1-130 and *L. interrogans* Serovar Lai Strain 56601

**Figure 1:**
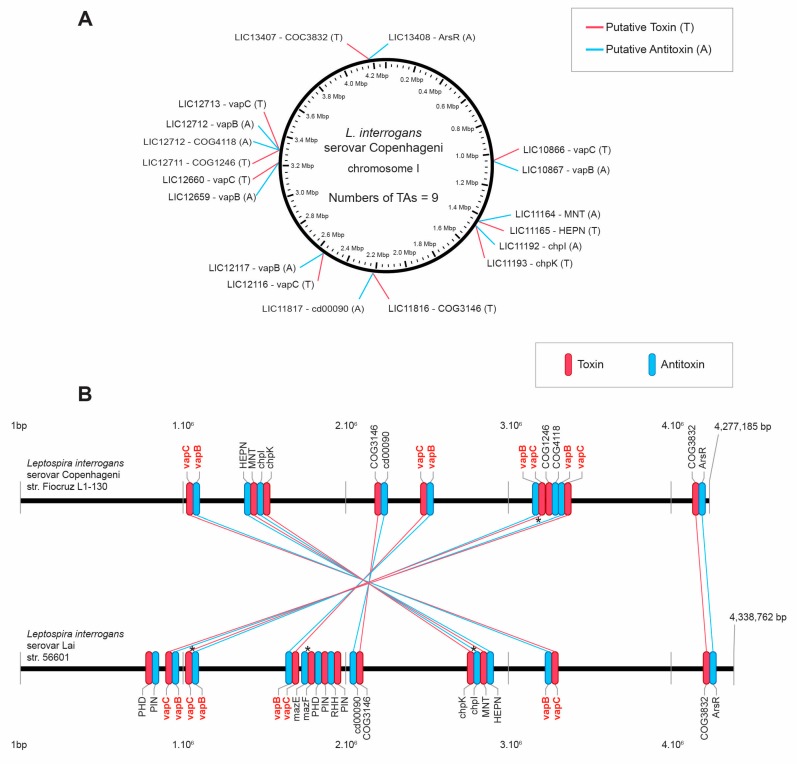
(**A**) Location map of toxin-antitoxin (TA) type II systems on the chromosome of *L. interrogans* serovar Copenhageni. (**B**) Comparison of the TAs sets of *L. interrogans* serovar Copenhageni and *L. interrogans* serovar Lai. In this schema, horizontal black lines represent the chromosomes of Copenhageni and Lai serovars. Red and blue lines indicate the amino acid sequence correspondence of toxins and antitoxins, respectively. Identity between linked toxins is greater than 90%. Toxins and antitoxins are identified by family or domains, as they appear in TAfinder. * denotes TA modules that were experimentally characterized and published.

Investigation of type II TA modules in the genome of *L. interrogans* serovar Copenhageni strain Fiocruz L1-130 and *L. interrogans* serovar Lai strain 56601 by the TAfinder tool of TADB resulted in the identification of nine modules in the strain Fiocruz L1-130 (Figure 1A) coding for four VapBC, one MazEF (ChpIK), and four TA modules of unclassified families (according TADB), in which the domains are: HEPN/MNT, cd00090/COG3146, COG4118/COG1246, and ArsR/COG3832. The *L. interrogans* serovar Lai strain 56601 was shown to have all the operons identified in the strain Fiocruz L1-130, except for the TA pair LIC12711/12712. In addition, the serovar Lai strain presented three TA modules, one MazEF, and two extra possible VapBC, inserted in the genome between the modules 4 and 5 of the *L. interrogans* serovar Copenhageni. The large chromosomal inversion described in the genome of the two *L. interrogans* strains investigated [44] can be observed by the inverted position of seven TAs of the serovar Copenhageni in relation to serovar Lai, except for the last TA of both chromosomes LIC 13407/13408–LA4258/4259 that are parallelly located (Figure 1B).

#### 3.1.2. *L. borgpetersenii* Serovar Hardjo-bovis Strain JB197 and Strain L550

Search by TAfinder and BLASTp comparison identified nine conserved TA modules in both strains of *L. borgpetersenii* analyzed here. They code for three VapBC, one MazEF, and five TA modules of unclassified families, in which the domains are: HEPN/MNT, PRK13696/cd09981, cd00090/COG3146, RHH/COG2929, and ArsR/COG3832. TAs of both *L. borgpetersenii* strains are displayed in completely inverted positions in their genomes, as shown in supplementary results (Table S1).

#### 3.1.3. *L. biflexa* Serovar Patoc Strain Patoc 1 (Ames) and Strain Patoc 1 (Paris)

The same analysis (TAfinder and BLASTp) of TAs of the genomes of *L. biflexa* resulted in the identification of 23 conserved TA modules. They code for eight VapBC, two MazEF, six RelBE, one phd/doc, and six TA modules of unclassified families, in which the domains are: HEPN/MNT, RHH-COG2929, and four ArsR/COG3832. The TA positionings in the genomes of both strains studied here are fully parallel (Table S2).

### 3.2. Variability of Amino Acids Sequences of VapCs within Leptospira Strains

Based on the fact that the toxins of TA modules are the elements responsible for the function and substrate specificity, and also for the family classification of these systems, we have focused this study on the distribution and conservation of the VapC toxins, from the VapBC family, which are known to have poor conservation of the primary sequences but high conservation of the PIN domain structural fold [45].

**Figure 2:**
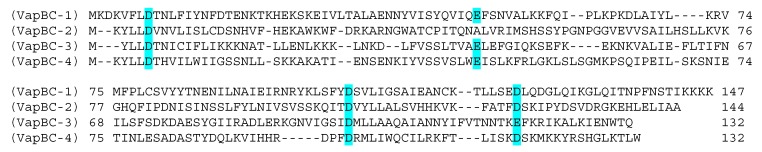
Alignment of the VapCs of *L. interrogans* serovar Copenhageni. Alignment was done using the COBALT tool (Constraint-based Multiple Alignment Tool), which aligns the sequences via considering the amino acid sequence and protein domains. The numbers of VapCs in parentheses are based on the numerical order of the genes in the chromosome and refer to the loci described in Table 1. The highlighted acidic amino acids comprise the residues essential to the catalytic activity and classification within the PIN (PilT N-terminal) domain.

According to the search conducted on TAfinder, the *L. interrogans* serovar Copenhageni strain Fiocruz L1-130 has four vapBC loci, all on chromosome I, which encode four toxin-antitoxin modules of the VapBC family. Based on the numerical order of the genes in the chromosome, only VapBC-3 (LIC12660–12659) was experimentally characterized and thus considered a bona fide element [29], while the others remain to be elucidated. The alignment of the amino acid sequences of these four VapCs (Figure 2) shows the diversity between their primary sequences, but with conservation of the set of three or four acid residues responsible for coordinating Mg^2+^ or Mn^2+^ ions at the catalytic site. The cognate VapB antitoxins partners comprise different types of protein domains (RHH, AbrB, and PHD) and therefore cannot have their primary structures compared.

In order to investigate whether the amino acid sequences of the toxins from the VapBC modules are divergent within their own bacterial strain, we have evaluated the VapCs’ identities using the tool “Global Align” for the four VapCs of *L. interrogans* serovar Copenhageni Fiocruz L1-130, the 15 VapCs *L. licerasiae* serovar Varillal strain VAR010, and the eight VapCs *L. biflexa* serovar Patoc 1 (Ames) (Table 1, Figure S1). The analysis of the sequence identities within each of the strains were found to be mostly very low, varying from 13% to 46%, with most of them being around 20%, corroborating the well-known low amino-acid identity among VapC toxins.

### 3.3. Distribution of VapCs of Pathogenic, Intermediate, and Saprophytic Leptospira Strains

In order to study how the amino acid sequences of VapCs are conserved among leptospiral pathogenicity groups (pathogenic, intermediate, and saprophytic) and to infer functional and evolutionary relationships between sequences, we have carried out an extensive study using the BLAST tool, in which we submitted the identified VapCs sequences of two pathogenic strains (*L. interrogans* and *L. borgpetersenii*), one intermediate strain (*L. licerasiae*), and one saprophytic strain (*L. biflexa*) to compare with sequence database of the *Leptospira* taxid for each of the species described in the methods section. To enable this comparison, we developed a parameter to indicate “conservation” between sequences named *C-value* (*Cv*) (see the methods section), which takes into account three results provided by BLAST analysis: the coverage (“*c*”), the number of identical amino acids (“*i*”), and also the number of conserved amino acids designed as positive (“*p*”), which varies from 0 to 1. We did not use the e-value given by BLAST because it varies according to protein size and it does not consider conservative mutations, which are known to be important to structure and function, as previously indicated for VapC-3 [29]. We present here the results comparing the four strains mentioned above. All tables were colored to indicate, through color intensity, the degree of conservation among the toxins evaluated by *C-value*. Results for *L. interrogans* serovar Copenhageni are shown in Table 2. Detailed results for the remaining three strains are shown in Tables S3–S5. In addition to the *C-values*, the results also include the “*i*”, “*p*”, and “*c*” values. It is important to consider that BLAST searches for similar sequences based on a large number of entries from several isolates and that each of the analyzed species have (for the most part) many entries too, and that some of the entries of specific strains do not have the serovar group defined. With that said, it should be noted that when one cell of the Table 2, Table 3, Table 4 and Table 5 shows that a particular VapC is present in a given species (e.g., *L. interrogans* or *L. noguchii*), it is not necessarily present in all members of this species, but in one or some isolates of them.

#### 3.3.1. *L. interrogans* Serovar Copenhageni Fiocruz L1-130

BLAST analysis of the four VapC sequences showed that VapC-3, previously described and characterized [29], is the toxin most widely distributed, with high *C-value*, among the 20 *Leptospira* species studied, followed by VapC-4 (Table 2). In contrast, VapC-1 amino acid sequence shows high *C-value* only for the pathogenic species *L. kirschneri* and *L. alexanderi*. Interestingly, VapC-2 is the only one of the four toxins that is highly conserved exclusively among most of the pathogenic species with the exception of *L. borgpetersenii* and *L. kmetyi* species. It should be noted that these two pathogenic species only showed conservation within the VapCs of *L. interrogans* serovar Copenhageni Fiocruz L1-130.

In parallel, we evaluated the similarity of these four VapCs among 12 other important human pathogenic bacteria species, such as *Mycobacterium tuberculosis* and *Shigella sonnei* (see Supplementary Material—Table S6), but no conclusions could be taken from it. Interestingly, as for the *Leptospira* species, VapC-3 is the toxin most distributed among investigated bacteria, followed by VapC-4. VapC-1 and VapC-2 showed medium conservation only with *P. aeruginosa* and *M. tuberculosis*.

#### 3.3.2. *L. borgpetersenii* Serovar Hardjo-Bovis Strain JB197

We also analyzed the conservation of the VapCs of pathogenic *L. borgpetersenii* (Table 3), whose genome was completely sequenced. From the nine TA modules identified by TAfinder, three belong to the VapBC family. VapC1 shows the highest distribution among the species analyzed, concentrating high *C-value* among the pathogenic species. Similar to VapC-2 of *L. interrogans* serovar Copenhageni, *L. borgpetersenii* VapC-2 is highly conserved only among pathogenic species. VapC-3 amino acid sequence is highly conserved only in *L. weilii*, with diffuse distribution and low *C-value* among others.

#### 3.3.3. *L. licerasiae* Serovar Varillal strain VAR010

Among the 28 TAs identified in the intermediate pathogenic *L. licerasiae* [39], 15 are VapBC modules (Table 4). We observed a diffuse pattern of conservation to the toxins. VapCs 1, 2, 4, and 10 appear with low *C-values* among most species, while VapCs 3, 5, 7, and 8 exhibit wide distribution and medium conservation *C-values*. VapCs 6, 9, 11, 13, and 14 present a larger distribution with high *C-values* indicating higher conservation of sequences. It should be noted that VapCs 8, 12, and 15 (LEP1GSC185:2580, LEP1GSC185:3559, and LEP1GSC185:3880) share the same amino-acid sequence, probably stemming from genetic duplication events.

#### 3.3.4. *L. biflexa* Serovar Patoc 1 (Ames)

When the nonpathogenic *L. biflexa* strain was analyzed (Table 5), we observed a more homogenous pattern of distribution (i.e., we noted that, with the exception of VapC1, all the others displayed high *C-values* in the other saprophytic strains, and the VapCs 4, 5, 6, 7, and 8 appear with relatively high conservation in the pathogenic species as well). It is interesting to note that among the eight VapCs of *L. biflexa*, three of them (VapC-4, VapC-5, and VapC-6) resemble VapC-3 of *L. interrogans* Copenhageni, which might suggest some evolutionary link and explain their strong presence among the pathogenic species.

Taken all together, our results describing the distribution of these TA modules among leptospiral strains of varied phenotypes of pathogenicity showed that VapCs are unequally distributed, with some toxins apparently being randomly disseminated, to others present in unique species or specific pathogenicity groups.

## 4. Discussion

Toxin-antitoxin systems have been involved in potentially harmful aspects of an infection, such as antimicrobial resistance, persistence, and biofilm formation, and therefore have been the subject of intensive efforts to elucidate biochemical and functional effects besides their role on the physiology of infection. Nevertheless, due to the high number of types, families, and high divergence among them, all these efforts on the elucidation of biochemical and biological functions are still incipient and remain controversial.

Currently, three type II TAs were described and experimentally characterized in the genome of *L. interrogans* serovar Copenhageni strain Fiocruz L1-130 and serovar Lai strain Lai: one VapBC [29,46], one ChpIK, and one MazEF [9,47], and therefore can be considered bona fide elements, all the others need to be confirmed. It should be pointed out that the ChpKI module has also been called as MazEF [48] since both ChpI and MazF toxins share the structurally and functionally similar domain PemK, which is a sequence-specific endoribonuclease [49]. Even though both *chp*IK and *maz*EF, found in *L. interrogans* serovar Lai strain Lai, have been described as extensively distributed and the sequences conserved in different pathogenic *Leptospira* [9], the *maz*EF operon (LA1780/1781) was not identified in the *L. interrogans* serovar Copenhageni strain Fiocruz L1-130 using BLAST analysis, but it is present only in the *L. interrogans* serovar Copenhageni strain HAIO156 from the 19 isolates available of this species-serovar. Considering the strict curation of Fiocruz L1-130 and Lai genomes, this fact indicates that the function of MazEF in pathogenesis is not essential or, more likely, redundant, and could be replaced by other TAs in these strains.

The analysis of the whole genomes of *Leptospira* has shown that there is considerable genomic plasticity even within the same species, as in the case of the large inversion in chromosome I and an ~54 kb genomic island that differentiates the genomes of *L. interrogans* serovars Lai and Copenhageni, which share ~99% similarity at amino acid level of ortholog genes [44]. Our data shows that the disposition of seven TAs along the chromosome of both bacteria is inverted as expected, but more significantly, have the addition of three TA modules. Similarly, we have found that the genomes of both strains of *L. borgpetersenii* analyzed (JB197 and L550) displayed total inversion of the seven TA sequences along the chromosome, which share exactly the same amino acid sequences (data not shown). Differently, the analysis of the 23 TA modules of the genomes of the two strains of *L. biflexa* (Ames and Paris) did not show any variation between them, even in their position on the chromosome, which is in agreement with data in the literature describing the high conservation in the genomes within this species [43].

The low intra-species amino acid sequence identity among VapCs of the *L. interrogans*, *L. licerasiae*, and *L. biflexa* strains evaluated in our study might indicate that they act on different substrates producing distinct effects, however, no link between toxin homologs and a specific physiological function has been established [50,51]. Additionally, it remains controversial whether such TA systems are redundant or not in relation to their physiological functions. It has been postulated that TA systems are responsive to environmental changes, both within their hosts or in free-living conditions. In the case of type II TAs, such changes would trigger the activity of enzymes such as Lon protease [52], which would cause antitoxin degradation, de-repressing transcription, increasing TA expression, and finally releasing the toxins to perform their functions. TAs have been proposed to participate in bacterial pathogenicity based on the idea that adaptation of pathogenic bacteria to the host is intrinsically linked to the expression of virulence genes. VapBC modules have been suggested to be involved with bacterial phenotypes both under intra-host and free-living conditions, which was shown by the loss of adaptability to stressing conditions when knocking out the operons *vapBC2ST* of *Salmonella typhimurium* [53], *vapBC-1* of *Haemophilus influenzae* [13], and *vapBC* of the thermophilic *Sulfolobus solfataricus* [54]. In *L. interrogans* Copenhageni, VapBC-3 has been biochemically characterized to act on the lysis of the initiator tRNA^fMet^ and therefore to inhibit translation [29], but information of its role in the physiology of the bacteria is still lacking.

TAs are represented by a highly variable number of modules in several bacteria, as highly variable as the extreme environmental or host differences to which these organisms must adapt to live, suggesting it might contribute to their diversification and evolution. Unsurprisingly, the resulting phenotype of each species is affected by a diversity of features including the whole genomic background, among which the diversity of TA modules can be included and may interfere in the response of individual TA systems during adaptation stresses [55]. In the case of *Leptospira*, the overall set of TA modules characteristic among species living in saprophytic or pathogenic conditions reveals that the *L. biflexa* has a considerable larger number of TAs then *L. interrogans* and *L. borgpetersenii*, which can indicate a relatively large subset of adaptive facilities. However, the intermediate *L. licerasiae*, whose genomic analysis led to the conclusion of its greater proximity to pathogenic strains than to saprophytic [39], has an even greater set of TA systems, in general, and *vap*BC specifically. Although the data available to date are very incipient, we could hypothesize that this larger number of modules found in the intermediate pathogenic *L. licerasiae*, which could also be referred as “intermediate saprophytic” [56], would be related to its ability to survive in both the environment and animal hosts. There are examples in the literature showing that proposed TAs, exhibiting a typical TA genetic organization (e.g., XreA-Ant and Bro-XreB of *S. pneumonia* [57] and VapBC of *M. tuberculosis* [58]), did not appear to act as bona fide TAs when experimentally tested. Therefore, it is important to reinforce that the results and hypothesis discussed here are based on in silico analysis and questions related to the possible redundancy and functionality of the VapBC modules analyzed here need to be confirmed by in vivo experiments.

It is not clear how acquisition by horizontal gene transfer of the set of TA loci influenced the evolution of each bacterial species in its broad set of genes, and whether TAs may play roles in determining phenotypic aspects such as virulence and persistence [14,51]. It has been suggested that TAs should more likely be considered as just selfish elements that would improve the fitness of bacteria to eventual stressing conditions, driving the evolution of bacterial genomes [21].

By providing an overview of the distribution of *vap*BC operons among *Leptospira* species, through the analysis of the conservation of their toxic elements, we could outline a rather complex picture indicating that these modules evolved differently. It is remarkable that, out of all VapC sequences of pathogenic species submitted to BLAST analysis, VapC-2 of *L. interrogans* serovar Copenhageni and VapC-2 of *L. borgpetersenii* serovar Hardjo-bovis are, at the same time, widely and strictly highly conserved only among pathogenic species. Interestingly, proteomic analysis showed an increase of the antitoxin component from VapBC-2 of *L. interrogans* serovar Copenhageni during stress induced by antibiotic treatment (Ciprofloxacin) [59]. Thus, they might represent potential candidates for participating in the adaptation of the pathogen to the infection host and have a role in pathogenesis.

Even though in recent years we have accumulated an increase in the knowledge about TA systems, information on how they have evolved is still missing. One reason that may help to explain this lack of knowledge is the fact that most TA loci are acquired by horizontal gene transfer [39,60] and thus are often not conserved in different isolates belonging to the same bacterial species. According to that explanation and corroborating our results, a study dealing with dissemination type II TAs in *Escherichia coli* showed that they are unevenly distributed among *E. coli* phylogroups and are not a universal feature of the species [61]. In parallel studies, our attempts at using molecular phylogenetic analysis to try to identify some branching points and thus infer some correlation of VapCs with pathogenic, intermediate, and saprophytic species of *Leptospira* has failed. Except in the specific cases mentioned above, our data were inconclusive and unable to infer this kind of correlation, most likely due to the high diversity of their amino acid sequences. Moreover, it is very important to consider that protein primary sequences are the groundings for the three-dimensional structure folding, which ultimately is responsible for toxin and antitoxin activities.

The large variability in numbers and the low sequence conservation among VapCs shown here emphasizes the strong need to identify and characterize new TAs as well to understand the regulation nets and roles of TA systems in pathogenic bacteria.

## Figures and Tables

**Table 1:** Amino acid sequence identities of VapCs within the same *Leptospira* strains.

**A*—L. interrogans* Serovar Copenhageni str. Fiocruz L1-130—Identity (%)**							**B—*L. biflexa* Serovar Patoc Strain Patoc 1 (Ames)—Identity (%)**										
		VapC-1	VapC-2	VapC-3	VapC-4				VapC-1	VapC-2	VapC-3	VapC-4	VapC-5	VapC-6	VapC-7	VapC-8	
			
VapC-1		100	19	23	18		VapC-1		100	17	13	24	19	19	27	21	
VapC-2			100	19	17		VapC-2			100	18	23	19	22	20	17	
VapC-3				100	26		VapC-3				100	27	22	16	16	19	
VapC-4					100		VapC-4					100	22	22	22	28	
							VapC-5						100	36	19	46	
							VapC-6							100	17	38	
							VapC-7								100	22	
							VapC-8									100	
**C*—L. licersiae* Serovar Varillal str. VAR 010—Identity (%)**																	
		VapC-1	VapC-2	VapC-3	VapC-4	VapC-5	VapC-6		VapC-7	VapC-8	VapC-9	VapC-10	VapC-11	VapC-12	VapC-13	VapC-14	VapC-15
	
VapC-1		100	26	18	19	28	17		22	14	24	18	17	14	23	21	14
VapC-2			100	13	19	31	21		18	17	19	16	19	17	20	21	17
VapC-3				100	21	14	17		19	17	20	17	24	17	22	23	17
VapC-4					100	18	21		28	25	21	18	22	25	20	31	25
VapC-5						100	23		18	21	24	20	18	21	21	19	21
VapC-6							100		16	18	32	19	16	18	21	21	18
VapC-7									100	32	18	19	18	32	18	22	32
VapC-8										100	23	19	20	100	15	23	100
VapC-9											100	17	20	23	19	25	23
VapC-10												100	20	19	12	21	19
VapC-11													100	20	20	17	20
VapC-12														100	15	23	100
VapC-13															100	20	15
VapC-14																100	23
VapC-15																	100

(**A**) VapCs of *L. interrogans* serovar Copenhageni Fiocruz L1-130. (**B**) VapCs of *L. biflexa* serovar Patoc 1 (Ames). (**C**) VapCs of *L. licerasiae* serovar Varillal strain VAR010.

**Table 2:** Basic Local Alignment Search Tool (BLAST) analysis of VapCs of *L. interrogans* serovar Copenhageni Fiocruz L1-130.

VapC of *L. interrogans* Serovar Copenhageni str. Fiocruz L1-130																	
*Leptospira* spp.		LIC10866 VapC-1				LIC12116 VapC-2				LIC12660 VapC-3				LIC12713 VapC-4			
		Identity (%)	Positives (%)	Cover (%)	*C-value*	Identity (%)	Positives (%)	Cover (%)	*C-value*	Identity (%)	Positives (%)	Cover (%)	*C-value*	Identity (%)	Positives (%)	Cover (%)	*C-value*
Pathogenic	*L. interrogans* *L. interrogans*	100	100	100	1.00	100	100	100	1.00	100	100	100	1.00	100	100	100	1.00
	*L. kirschneri*	99	99	100	0.99	97	98	100	0.98	50	66	97	0.56	97	97	100	0.97
	*L. noguchii*	-	-	-	-	97	99	100	0.98	44	64	97	0.52	-	-	-	-
	*L. borgpetersenii*	-	-	-	-	-	-	-	-	48	68	97	0.56	-	-	-	-
	*L. weilii*	-	-	-	-	87	95	97	0.88	90	95	100	0.93	48	66	100	0.57
	*L. santarosai*	-	-	-	-	83	90	100	0.87	66	83	100	0.75	64	75	100	0.70
	*L. alexanderi*	93	95	100	0.94	85	94	100	0.90	89	94	100	0.92	-	-	-	-
	*L. alstoni*	-	-	-	-	86	95	100	0.91	90	96	100	0.93	61	73	97	0.65
	*L. kmetyi*	-	-	-	-	-	-	-	-	-	-	-	-	-	-	-	-
Intermediary	*L. wolffii*	-	-	-	-	-	-	-	-	44	68	97	0.54	45	62	100	0.54
	*L. licerasiae*	-	-	-	-	-	-	-	-	70	87	99	0.78	44	60	100	0.52
	*L. inadai*	-	-	-	-	-	-	-	-	63	84	99	0.73	65	78	100	0.72
	*L. fainei*	-	-	-	-	-	-	-	-	-	-	-	-	-	-	-	-
	*L. broomii*	-	-	-	-	-	-	-	-	-	-	-	-	63	75	100	0.69
Saprophytic	*L. wolbachii*	-	-	-	-	-	-	-	-	66	83	99	0.74	64	75	100	0.70
	*L. meyeri*	44	70	95	0.54	-	-	-	-	66	84	99	0.74	62	74	100	0.68
	*L. biflexa*	-	-	-	-	-	-	-	-	62	82	99	0.71	28	55	93	0.39
	*L. vanthielii*	46	69	95	0.55	-	-	-	-	51	69	98	0.59	61	75	100	0.68
	*L. terpstrae*	-	-	-	-	-	-	-	-	-	-	-	-	-	-	-	-
	*L. yanagawae*	-	-	-	-	-	-	-	-	48	70	98	0.58	-	-	-	-

*Conservation values* (*C-values*) were expressed as a frequency between 0 and 1. The colors indicate: 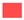 very highly conserved (0.85 ≤ *C-value* ≤ 1.0); 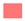 highly conserved (0.7 ≤ *C-value* ≤ 0.84); 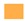 moderately conserved (0.4 ≤ *C-value* ≤ 0.69); 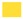 poorly conserved (*C-value* ≤ 0.39); 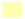 no hits. Positive and cover values below 50% were not included.

**Table 3:** Conservation analysis of VapCs of *L. borgpetersenii* serovar Hardjo-bovis strain JB197.

VapC of *L. borgpetersenii* Serovar Hardjo-bovis str. JB197—*Conservation value (C-value)*				
*Leptospira* spp.		LBJ_0624 VapC-1	LBJ_0764 VapC-2	LBJ_2077 VapC-3
Pathogenic	*L. interrogans* *L. interrogans*	0.95	0.90	0.39
	*L. kirschneri*	0.49	0.89	0.38
	*L. noguchii*	0.93	-	0.39
	*L. borgpetersenii*	1.00	1.00	1.00
	*L. weilii*	0.94	0.89	0.96
	*L. santarosai*	0.49	0.87	0.40
	*L. alexanderi*	0.52	0.48	-
	*L. alstoni*	0.95	0.88	0.44
	*L. kmetyi*	-	-	-
Intermediary	*L. wolffii*	0.49	-	-
	*L. licerasiae*	0.49	-	0.43
	*L. inadai*	-	-	0.38
	*L. fainei*	-	-	-
	*L. broomii*	-	-	-
Saprophytic	*L. wolbachii*	0.51	-	0.38
	*L. meyeri*	0.83	-	-
	*L. biflexa*	0.53	-	0.38
	*L. vanthielii*	0.82	0.47	0.38
	*L. terpstrae*	-	-	0.35
	*L. yanagawae*	0.51	-	-

*Conservation values* (*C-values*) were expressed as a frequency between 0 and 1. The colors indicate: 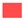 very highly conserved (0.85 ≤ *C-value* ≤ 1.0); 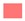 highly conserved (0.7 ≤ *C-value* ≤ 0.84); 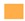 moderately conserved (0.4 ≤ *C-value* ≤ 0.69); 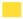 poorly conserved (*C-value* ≤ 0.39); 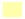 no hits. Positive and cover values below 50% were not included.

**Table 4:** Conservation analysis of VapCs of *L. licerasiae* serovar Varillal strain VAR010.

VapC of *L. licerasiae* Serovar Varillal str. VAR 010—*Conservation value* (*C-value*)															
*Leptospira* spp.		LEP1GSC185_0307—VapC-1	LEP1GSC185_0418—VapC-2	LEP1GSC185_0630—VapC-3	LEP1GSC185_1922—VapC-4	LEP1GSC185_2251—VapC-5	LEP1GSC185_2580—VapC-6	LEP1GSC185_3193—VapC-7	LEP1GSC185_3530—VapC-8 *	LEP1GSC185_3550—VapC-9	LEP1GSC185_3553—VapC-10	LEP1GSC185_3557—VapC-11	LEP1GSC185_3561—VapC-13	LEP1GSC185_3566—VapC-14
Pathogenic	*L. interrogans* *L. interrogans*	0.48	0.36	0.66	-	0.53	0.80	0.54	0.64	0.78	-	0.87	0.82	0.92
	*L. kirschneri*	-	-	0.66	-	0.53	0.41	0.41	0.64	0.56	-	0.82	-	0.90
	*L. noguchii*	-	-	0.65	-	0.20	0.46	0.42	0.63	-	-	0.54	0.85	0.92
	*L. borgpetersenii*	-	-	0.48	-	0.20	0.79	0.46	0.67	0.55	-	-	-	0.92
	*L. weilii*	-	-	0.48	-	0.67	0.80	0.45	0.66	0.93	0.91	0.86	-	0.77
	*L. santarosai*	-	0.37	0.46	-	0.54	-	0.42	0.63	0.89	-	0.54	0.94	0.72
	*L. alexanderi*	-	-	0.65	-	0.20	0.80	0.41	0.64	0.78	-	0.86	-	-
	*L. alstoni*	0.65	0.40	0.48	-	0.50	0.79	0.75	0.66	0.78	-	0.87	-	0.77
	*L. kmetyi*	-	-	-	-	-	-	-	-	-	-	-	-	0.92
Intermediary	*L. wolffii*	0.38	0.78	-	0.81	0.80	0.42	0.41	-	0.53	-	0.96	-	0.97
	*L. licerasiae*	1.00	1.00	1.00	1.00	1.00	1.00	1.00	1.00	1.00	1.00	1.00	1.00	1.00
	*L. inadai*	0.77	0.38	-	-	0.55	-	0.39	0.87	0.88	-	0.94	-	0.91
	*L. fainei*	-	-	-	-	-	-	-	-	-	-	0.93	-	-
	*L. broomii*	-	0.41	-	-	0.56	-	-	0.40	-	-	-	-	-
Saprophytic	*L. wolbachii*	-	0.39	-	-	0.52	0.42	0.42	0.64	0.85	-	0.81	-	0.37
	*L. meyeri*	-	0.38	0.65	-	0.54	0.78	0.41	0.64	0.85	-	0.55	-	0.73
	*L. biflexa*	-	0.42	-	-	0.37	0.77	0.40	0.66	0.80	-	-	-	0.73
	*L. vanthielii*	-	0.39	0.61	-	0.53	0.78	0.41	0.65	0.56	-	0.54	0.75	0.37
	*L. terpstrae*	-	-	-	-	-	-	0.41	0.66	-	-	-	-	-
	*L. yanagawae*	-	-	-	-	-	0.42	-	-	0.56	-	0.55	0.74	-

*Conservation values* (*C-values*) were expressed as a frequency between 0 and 1. The colors indicate: 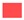 very highly conserved (0.85 ≤ *C-value* ≤ 1.0); 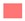 highly conserved (0.7 ≤ *C-value* ≤ 0.84); 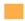 moderately conserved (0.4 ≤ *C-value* ≤ 0.69); 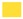 poorly conserved (*C-value* ≤ 0.39); 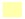 no hits. Positive and cover values below 50% were not included. * VapCs 8, 12, and 15 (LEP1GSC185:2580, LEP1GSC185:3559, and LEP1GSC185:3880) share the same amino-acid sequence. VapC-12 and VapC-15 were not shown here.

**Table 5:** Conservation analysis of VapCs of *L. biflexa* serovar Patoc 1 (Ames).

VapC of *L. biflexa* serovar Patoc strain Patoc 1 (Ames)—*Conservation value (C-value)*									
*Leptospira* spp.		LBF_0418 VapC-1	LBF_2142 VapC-2	LBF_ 2175 VapC-3	LBF_2183 VapC-4	LBF_2185 VapC-5	LBF_2276 VapC-6	LBF_2813 VapC-7	LBF_3285 VapC-8
**Pathogenic**	*L. interrogans* *L. interrogans*	0.40	0.60	-	0.78	0.72	0.83	0.83	0.59
	*L. kirschneri*	0.40	-	-	0.78	0.55	-	0.83	0.73
	*L. noguchii*	-	0.61	-	0.77	0.48	0.47	0.54	0.52
	*L. borgpetersenii*	0.35	-	0.27	0.78	0.55	0.89	-	0.73
	*L. weilii*	0.42	0.59	0.60	0.78	0.83	0.89	0.83	0.73
	*L. santarosai*	0.41	0.62	-	0.78	0.78	0.55	0.53	0.75
	*L. alexanderi*	0.35	0.59	0.60	0.77	0.72	0.88	0.84	0.72
	*L. alstoni*	-	-	0.59	0.79	0.72	0.85	0.83	0.73
	*L. kmetyi*	-	-	-	-	-	-	-	-
**Intermediary**	*L. wolffii*	-	-	-	-	0.52	0.44	0.80	0.75
	*L. licerasiae*	0.44	-	-	0.56	0.80	0.79	0.82	0.55
	*L. inadai*	0.41	-	-	0.63	0.79	0.33	0.80	0.50
	*L. fainei*	-	-	-	-	-	-	0.79	-
	*L. broomii*	0.42	-	-	-	-	-	-	-
**Saprophytic**	*L. wolbachii*	0.40	0.94	0.99	0.88	0.92	0.45	0.87	0.92
	*L. meyeri*	0.43	0.95	0.96	0.96	0.84	0.97	0.52	0.91
	*L. biflexa*	1.00	1.00	1.00	1.00	1.00	1.00	1.00	1.00
	*L. vanthielii*	0.43	0.95	-	0.91	0.56	0.97	0.53	0.93
	*L. terpstrae*	-	-	-	0.91	-	-	-	-
	*L. yanagawae*	-	0.95	0.97	-	0.57	0.44	0.53	0.95

*Conservation values* (*C-values*) were expressed as a frequency between 0 and 1. The colors indicate: 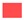 very highly conserved (0.85 ≤ *C-value* ≤ 1.0); 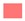 highly conserved (0.7 ≤ *C-value* ≤ 0.84); 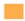 moderately conserved (0.4 ≤ *C-value* ≤ 0.69); 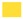 poorly conserved (*C-value* ≤ 0.39); 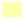 no hits. Positive and cover values below 50% were not included.

## Supplementary Materials

The following are available online at http://www.mdpi.com/2076-2607/7/2/56/s1.
