# Supplementary material for: In Silico Analysis of Genetic VapC Profiles from the Toxin-Antitoxin Type II VapBC Modules among Pathogenic, Intermediate, and Non-Pathogenic Leptospira
Source: Microorganisms. 2019 Feb 20;7(2):56. doi: 10.3390/microorganisms7020056 (PMC6406750; doi:10.3390/microorganisms7020056)
Supplement: Supplementary file 1 [file microorganisms-07-00056-s001.pdf]

Figure S1

***Leptospira interrogans* serovar Copenhageni str. Fiocruz L1-130**

>LIC-VapC1

MKDKVFLDTNLFIYNFDTENKTKHEKSKEIVLTALAENNYVISYQVIQEFSNVALKKFQIPLKPKDLAIY
LKRVMFPLCSVYYTNENILNAIEIRNRYKLSFYDSVLIGSAIEANCKTLLSEDLQDGLQIKGLQITNPFN
STIKKKK

>LIC-VapC2

MKYLLDVNVLISLCDSNHVFHEKAWKWFDRKARNGWATCPITQNALVRIMSHSSYPGNPGGVEVVSAILH
SLLKVKGHQFIPDNISINSSLFYLNIVSVSSKQITDVYLLALSVHHKVKFATFDSKIPYDSVDRGKEHLE
LIAA

>LIC-VapC3

MYLLDTNICIFLIKKKNATLLENLKKKLNKDLFVSSLTVAELEFGIQKSEFKEKNKVALIEFLTIFNILS
FSDKDAESYGIIRADLERKGNVIGSIDMLLAAQAIANNYIFVTNNTKEFKRIKALKIENWTQ

>LIC-VapC4

MKYLLDTHVILWIIGSSNLLSKKAKATIENSENKIYVSSVSLWEISLKFRLGKLSLSGMKPSQIPEILSK
SNIETINLESADASTYDQLKVIHHRDPFDRMLIWQCILRKFTLISKDSKMKKYRSHGLKTLW

***Leptospira borgpetersenii* serovar Hardjo-bovis str. JB197**

>LBJ_0624-VapC1

MNYFLDTNICIYFLKGKGENIEKNIRKLNPNRIKIPSVVKAELLLGAFKSNDQKKNRESVLSFLDPFEII
GFNDIESEIYAEVRSGLEIQGIPIGPNDLLVASVVLSSNGILVTNNEKEFKMIPNLKIENWL

>LBJ_0764-VapC2

MPVYIDTSFFLSIIFEDTNYELSYESWIGDDYRFSSSLLGIESFVNIHKVYRENRKVLNKRWLDESLTRQ
RELLTVINLKRIGSEVYEKIQKTEKLTFLKSLDSII

>LBJ_2077-VapC3

MRIVLDANVIISALISNGLSHQVVDICIDDHEVFMSKALNTEVLNFFVRKVKTTIDEKHRIAKFLDELGP
EIVPKGKPPVICRDKNDNHVLHLVQTCSARILISGDQDLLVLKKHEHTPILTPRQFLEVLNSGKLDGENF
A

***Leptospira biflexa* serovar Patoc strain Patoc 1 (Ames)**

>LBF-VapC1

MKYLLDTQIFIFALENPTMIPPKIRKVLESFESELFVSDVSVWEMIIKASIKKLKFKSDIKQVITKGYDV
LGANDLLIQKSHIFRSMTLPFHHKDPFDRLLASQALEEDLLFLTTDVIFKKYKVKVL

>LBF-VapC2

MSYLIDTDIILYSLKGNEKVQNNLIEKKNISKVISVITYGELIFGAKKSKSREKNLATVYRIGELFPVIE
LTKGIVETFGEVKATVQKKGNTVDDFDLLIGSTALFLNYTLVTNNEKHFSLIPDLKIENWSK

>LBF-VapC7

MKNVALIDSGPIIALFNEKDKFHKSILKYLKSFKGELISSWPVVTEVIYLLSFSVEAQSDFLEWIERGGI
QIFNLSIEDLKYIKSRMKKYSDLPMDLADASLMCIAEKFEYENIISIDSDFSIYKTVKGKFLRNLYHV

>LBF-VapC8

MNHYLLDTNICIYIINKKPENVYQKFKKISLDNIFISSITEFELRYGVQKSQKADKNQKTLNEFLGYLNV
INFDSESASVAGTIRTKLEKKGEIIGPYDLLIASQAIASDIILVTNNEKEYKRIKELKIENWIN

***Leptospira licerasiae* serovar Varillal str. VAR 010**

>LEP1GSC185_0307-VapC1

MIVLDTHAWIWLMEGDPRMEKEPILRKLYRHIPHRGIFISEISGWEVGMLVAKKRIQISGTLNRWLQDAY
NAPGIQPYNLSPEVIVESVNLPDSFHGDPADRIIVATARVLNAELVTKDKEIIKYGKKGNLKVISL

>LEP1GSC185_0418-VapC2

MRTILLDTQIILWFLLEDPKLPPIIKELCKEEDTRLLFHQVSLWEIQIKYDLGKLPLPEMPGYFLVNACV
QSGLEKSAMQDEAIFFLTRLPNIHRDPFDRLLLSHAMINGWEFATTDEILKKYPVRILENIKT

>LEP1GSC185_0630-VapC3

MVLYIDTSLLLNILYAEAGYQDHLDYFNRSDLKFGSILLEIESFRSLHFTYSKEGKSLPKTWFKDAESFL
GEFISQINLKNLDADIRTEIQKNKEVLELKSLDAAHLATALHIRKSISDDLILCSMDEKFRSVAKKMGFK
LYPKK

>LEP1GSC185_1922-VapC4

MTAVLVDTSVWINHLRRSDPKLVELLHQGLVRRHPMVEGELSLGNFKNKNSFLTEYALLKEVPIANHKET
MIFSERNSLAGLGIGWIDAH LLASCILGSAKLYSADLSLVKAAEKIGIAEITT

>LEP1GSC185_2251-VapC5

MNYLLDTHAILWVLFQPENLSYKVESEILNHRNRIFISSISLWEISLKFSLRKIDLQGITPEQLPRKIRE
AGFEFIEDSPEIFANYFKLPTGKHTDPFDRFLVWQAVSFKFVFITKDRYLKEYNPLGLRLFW

>LEP1GSC185_2580-VapC6

MSYLIDTDIIIYSLKEDPIVRQNFLDRKNSIKSLSVITYGELIFGAQKSTYKERNLATVRRIAELFPVIQ
LTEGIMETYGELKAIQQKKGNTVEDFDLLIGSTALYLNYTLVTNNEKHFQKIPGLRMENWAYVT

>LEP1GSC185_3193-VapC7

MKIVLDTNVLLSSYLFQGYTAEVFDHVWLNHEIILSEWILTEFKEVCSRKFKIKEVDIREVLDHLRGGAK
VYQPKGRPPKVCADPDDDNILHIAEFSKSDWILSGDSDLLKLKQFQKIEIISPREYKLKFLV

>LEP1GSC185_3530-VapC8

MLKVLIDTNVYISAILFGGKPKVILEELISGKIIGYISDSILKEIEETLKKPKFKLSEEFISIVLSEIES
LTEKIVNISLKDYAGLRDRDDYHILESAISAKVDYLITGDQDLLVLKNLKSLKIISPEQYLSLDSSGASS
V

```
>LEP1GSC185_3561-VapC13

MNIVDSSGWLEYFAGTKRSALFSEAIEKTDKLFVPTISLYEVFKKIYLERDEDSALRAIAHMQQGTVIDL
DASISIFAAKLSKDHKIPMADSIILATARKYNAILWTQDDDFIGLDGVKYFPKK

>LEP1GSC185_3566-VapC14

MILVDTSVWIEFFRGNDPHFGELKELIESSEVIVHEVVFGELLQGCKNKTEVSFILEYWENLNTLTSDGS
FLLAGKLSFENKHIDKGIGLIDSVLINEVRSKKLQLWTLDKKILKVLDKKEIYSSRGKHVG

>LEP1GSC185_3880-VapC15

MLKVLIDTNVYISAILFGGKPKVILEELISGKIIGYISDSILKEIEETLKKPKFKLSEEFISIVLSEIES
LTEKIVNISLKDYAGLRDRDDYHILESAISAKVDYLITGDQDLLVLKNLKSLKIISPEQYLSLDSSGASS
V
```

Figure S1: Amino acid sequence of VapCs.

Table S1: Comparison of the TAs sets of *L. borgpetersenii* serovar Hardjo-bovis strain JB197 and strain L550

***Leptospira borgpetersenii* serovar Hardjo-bovis str. JB197 chromossome I**		***Leptospira borgpetersenii* serovar Hardjo-bovis str. L550 chromosome I**	
**TA Number**	**Location/Family/Domain**	**TA Number**	**Location/Family/Domain**
1	492171..492736/mazEF/AbrB-MazF	9	3169547..3170112 /mazEF/AbrB-MazF
2	754634..755259/vapBC /RHH-PIN	8	2907078..2907703/vapBC /RHH-PIN
3	921329..921957/vapBC/PHD-PIN	7	2740091..2740719/vapBC/PHD-PIN
4	1035975..1036644/ - /cd09981-PRK13696	6	1038972..1039641/vapBC/PRK13696-PIN
5	2250717..2252513/ - /COG3146-cd00090	5	1609875..16098441/ - /COG3146-cd00090
6	2448699..2448702/vapBC/RHH	4	113446..1135086/vapBC/RHH-PIN
7	3032705..3033037/ - /MNT-HEPN	3	641016..640990/ - /MNT-HEPN
8	3121041..3121588/ - /COG2929-RHH	2	552751..552754/ - /COG2929-RHH
9	327738..3237751/ - /COG3832-ArsR	1	403008..403788/ - /COG3832-ArsR

Table S2: Comparison of the TAs sets of *L. biflexa* serovar Patoc strain Patoc 1 (Ames) and strain Patoc 1 (Paris)

***L. biflexa* serovar Patoc strain Patoc 1 (Ames)**		***L. biflexa* serovar Patoc strain Patoc 1 (Paris)**	
**TA Number**	**Location/Family/Domain**	**TA Number**	**Location/Family/Domain**
1	208150..208954/ - /ArsR-COG3832	1	204108..204912/ - /ArsR-COG3832
2	428813..429605/ - /ArsR-COG3832	2	423243..424035/ - /ArsR-COG3832
3	432830..433456/ vapBC /PHD-PIN	3	427260..427886/ vapBC /PHD-PIN
4	652599..653253/ - /HEPN-MNT	4	647104..647683/ - /HEPN-MNT
5	762599..763164/ mazEF /AbrB-MazF	5	757029..757594/ mazEF /AbrB-MazF
6	2290126..2290820/ relBE /Xre-RelE	6	2284740..2285434/ relBE /Xre-RelE
7	2292479..2293160/ vapBC /COG2886-PIN	7	2287093..2287774/ vapBC /COG2886-PIN
8	2307062..2307723/ relBE /Xre-RelE	8	2301676..2302337/ relBE /Xre-RelE
9	2317508..2318144/ relBE /Xre-RelE	9	2312122..2312758/ relBE /Xre-RelE
10	2322690..2323354/ vapBC /RHH-PIN	10	2317304..2317968/ vapBC /RHH-PIN
11	2325925..2326536/ phd-doc /AbrB-Fic	11	2320539..2321150/ phd-doc /AbrB-Fic
12	2328778..2329439/ vapBC /RHH-PIN	12	2323392..2324053/ vapBC /RHH-PIN
13	2329722..2330350/ vapBC /AbrB-PIN	13	2324336..2324964/ vapBC /AbrB-PIN
14	2330621..2331281/ relBE /Xre-RelE	14	2325235..2325895/ relBE /Xre-RelE
15	2334551..2335149/ relBE /Xre-RelE	15	2329164..2329762/ relBE /Xre-RelE
16	2336628..2337332/ relBE /Xre-RelE	16	2331241...2331945/ relBE /Xre-RelE
17	2422710..2423445/ vapBC /RHH-PIN	17	2417379..2418114/ vapBC /RHH-PIN
18	2985341..2986203/ - /ArsR-COG3832	18	2976955..2977817/ - /ArsR-COG3832
19	3019939..3020593/ vapBC /RHH-PIN	19	3011553..3012207/ vapBC /RHH-PIN
20	3178445..3178762/ vapC/ PIN	20	3169797..3170385/ - /RHH-COG2929
21	3186177..3186756/ mazEF /RHH-MazF	21	3177800..3178379/ mazEF /RHH-MazF
22	3459352..3460666/ - /ArsR-COG3832	22	3450975..3452289/ - /ArsR-COG3832
23	3530394..3531025/ vapBC /AbrB-PIN	23	3522044..3522675/ vapBC /AbrB-PIN

Table S3: BLAST analysis of VapCs of *L. borgpetersenii* serovar Hardjo-bovis strain JB197.

VapC of *L. borgpetersenii* serovar Hardjo-bovis str. JB197													
***Leptospira* spp**		LBJ_0624 VapC-1				LBJ_0764 VapC-2				LBJ_2077 VapC-3			
		**Identity (%)**	**Positives (%)**	**Cover (%)**	***C-value***	**Identity (%)**	**Positives (%)**	**Cover (%)**	***C-value***	**Identity (%)**	**Positives (%)**	**Cover (%)**	***C-value***
**Pathogenic**	*L. interrogans*	93	96	100	0.95	88	94	99	0.90	33	54	90	0.39
	L. kirschneri	43	58	98	0.49	85	95	99	0.89	31	54	90	0.38
	L. noguchii	91	95	100	0.93	-	-	-	-	31	55	91	0.39
	L. borgpetersenii	100	100	100	1.00	100	100	100	1.00	100	100	100	1.00
	L. weilii	92	95	100	0.94	85	94	99	0.89	95	96	100	0.96
	L. santarosai	40	61	97	0.49	83	93	99	0.87	32	55	91	0.40
	L. alexanderi	46	59	99	0.52	91	98	51	0.48	-	-	-	-
	L. alstoni	94	96	100	0.95	85	93	99	0.88	33	56	99	0.44
	L. kmetyi	-	-	-	-	-	-	-	-	-	-	-	-
**Intermediary**	*L. wolffii*	39	62	98	0.49	-	-	-	-	-	-	-	-
	L. licerasiae	40	60	97	0.49	-	-	-	-	35	61	89	0.43
	L. inadai	-	-	-	-	-	-	-	-	28	52	94	0.38
	L. fainei	-	-	-	-	-	-	-	-	-	-	-	-
	L. broomii	-	-	-	-	-	-	-	-	-	-	-	-
**Saprophytic**	*L. wolbachii*	42	63	98	0.51	-	-	-	-	31	52	91	0.38
	L. meyeri	76	89	100	0.83	-	-	-	-	-	-	-	-
	L. biflexa	44	64	99	0.53	-	-	-	-	29	55	91	0.38
	L. vanthielii	75	88	100	0.82	37	57	99	0.47	30	53	91	0.38
	L. terpstrae	-	-	-	-	-	-	-	-	26	51	91	0.35
	L. yanagawae	42	62	98	0.51	-	-	-	-	-	-	-	-

Conservation values (*C-value*) were expressed as a frequency between 0 and 1. The colors indicate: very high conserved (0.85 ≤ *C-value* ≤ 1.0); high conserved (0.7 ≤ *C-value* ≤ 0.84); medium conserved (0.4 ≤ *C-value* ≤ 0.69); low conserved (*C-value* ≤ 0.39); no hits. Positives or cover values below 50% were discharged.

Table S4: BLAST analysis of VapCs of *L. licerasiae* serovar Varillal strain VAR010.

		VapC of *L. licerasiae* serovar Varillal str. VAR 010															
***Leptospira* spp**		LEP1GSC185_0307 - VapC-1				LEP1GSC185_0418 - VapC-2				LEP1GSC185_0630 - VapC-3				LEP1GSC185_1922 - VapC-4			
		Identity (%)	Positives (%)	Cover (%)	*C-value*	Identity (%)	Positives (%)	Cover (%)	*C-value*	Identity (%)	Positives (%)	Cover (%)	*C-value*	Identity (%)	Positives (%)	Cover (%)	*C-value*
Pathogenic	*L. interrogans*	48	74	79	0.48	31	50	89	0.36	58	76	99	0.66	-	-	-	-
	L. kirschneri	-	-	-	-	-	-	-	-	58	76	99	0.66	-	-	-	-
	L. noguchii	-	-	-	-	-	-	-	-	55	76	99	0.65	-	-	-	-
	L. borgpetersenii	-	-	-	-	-	-	-	-	38	61	97	0.48	-	-	-	-
	L. weilii	-	-	-	-	-	-	-	-	38	60	97	0.48	-	-	-	-
	L. santarosai	-	-	-	-	26	52	95	0.37	36	59	97	0.46	-	-	-	-
	L. alexanderi	-	-	-	-	-	-	-	-	57	75	99	0.65	-	-	-	-
	L. alstoni	55	77	98	0.65	31	53	96	0.40	38	61	97	0.48	-	-	-	-
	L. kmetyi	-	-	-	-	-	-	-	-	-	-	-	-	-	-	-	-
Intermediary	*L. wolffii*	30	51	93	0.38	73	89	96	0.78	-	-	-	-	79	86	98	0.81
	L. licerasiae	100	100	100	1.00	100	100	100	1.00	100	100	100	1.00	100	100	100	1.00
	L. inadai	69	84	100	0.77	28	53	95	0.38	-	-	-	-	-	-	-	-
	L. fainei	-	-	-	-	-	-	-	-	-	-	-	-	-	-	-	-
	L. broomii	-	-	-	-	32	54	96	0.41	-	-	-	-	-	-	-	-
Saprophytic	*L. wolbachii*	-	-	-	-	32	50	95	0.39	-	-	-	-	-	-	-	-
	L. meyeri	-	-	-	-	29	50	95	0.38	57	74	99	0.65	-	-	-	-
	L. biflexa	-	-	-	-	38	52	93	0.42	-	-	-	-	-	-	-	-
	L. vanthielii	-	-	-	-	30	53	95	0.39	51	75	97	0.61	-	-	-	-
	L. terpstrae	-	-	-	-	-	-	-	-	-	-	-	-	-	-	-	-
	L. yanagawae	-	-	-	-	-	-	-	-	-	-	-	-	-	-	-	-

***Leptospira* spp**		LEP1GSC185_2251 - VapC-5				LEP1GSC185_2580 - VapC-6				LEP1GSC185_3193 - VapC-7				LEP1GSC185_3530 - VapC-8*			
		Identity (%)	Positives (%)	Cover (%)	*C-value*	Identity (%)	Positives (%)	Cover (%)	*C-value*	Identity (%)	Positives (%)	Cover (%)	*C-value*	Identity (%)	Positives (%)	Cover (%)	*C-value*
Pathogenic	*L. interrogans*	45	61	100	0.53	77	87	97	0.80	60	79	77	0.54	60	78	93	0.64
	L. kirschneri	45	61	100	0.53	36	51	95	0.41	32	55	95	0.41	57	73	98	0.64
	L. noguchii	27	51	50	0.20	38	56	97	0.46	33	55	95	0.42	59	75	94	0.63
	L. borgpetersenii	27	51	50	0.20	76	87	97	0.79	35	61	95	0.46	61	78	97	0.67
	L. weilii	56	78	100	0.67	77	87	97	0.80	35	60	95	0.45	59	75	98	0.66
	L. santarosai	45	62	100	0.54	-	-	-	-	33	55	95	0.42	59	75	94	0.63
	L. alexanderi	27	51	50	0.20	77	87	97	0.80	33	54	95	0.41	60	76	94	0.64
	L. alstoni	44	59	97	0.50	76	86	97	0.79	66	84	100	0.75	60	74	98	0.66
	L. kmetyi	-	-	-	-	-	-	-	-	-	-	-	-	-	-	-	-
Intermediary	*L. wolffii*	73	87	100	0.80	35	53	95	0.42	51	80	62	0.41	-	-	-	-
	L. licerasiae	100	100	100	1.00	100	100	100	1.00	100	100	100	1.00	100	100	100	1.00
	L. inadai	47	63	100	0.55	-	-	-	-	31	52	94	0.39	83	93	99	0.87
	L. fainei	-	-	-	-	-	-	-	-	-	-	-	-	-	-	-	-
	L. broomii	50	62	100	0.56	-	-	-	-	-	-	-	-	35	52	93	0.40
Saprophytic	*L. wolbachii*	44	60	100	0.52	32	56	95	0.42	34	55	94	0.42	59	77	94	0.64
	L. meyeri	45	63	100	0.54	73	87	97	0.78	32	54	95	0.41	59	77	94	0.64
	L. biflexa	29	50	93	0.37	72	87	97	0.77	31	54	95	0.40	62	78	94	0.66
	L. vanthielii	43	63	100	0.53	73	87	97	0.78	32	55	95	0.41	62	77	94	0.65
	L. terpstrae	-	-	-	-	-	-	-	-	31	55	95	0.41	59	77	97	0.66
	L. yanagawae	-	-	-	-	35	54	95	0.42	-	-	-	-	-	-	-	-

***Leptospira* spp**		LEP1GSC185_3550 - VapC-9				LEP1GSC185_3553 - VapC-10				LEP1GSC185_3557 - VapC-11				LEP1GSC185_3561 - VapC-13				LEP1GSC185_3566 - VapC-14				***Leptospira* spp**	
		Identity (%)	Positives (%)	Cover (%)	*C-value*	Identity (%)	Positives (%)	Cover (%)	*C-value*	Identity (%)	Positives (%)	Cover (%)	*C-value*	Identity (%)	Positives (%)	Cover (%)	*C-value*	Identity (%)	Positives (%)	Cover (%)	*C-value*		
Pathogenic	*L. interrogans*	70	87	99	0.78	-	-	-	-	81	94	99	0.87	77	86	100	0.82	91	96	98	0.92	*L. interrogans*	Pathogenic
	L. kirschneri	50	66	97	0.56	-	-	-	-	76	90	99	0.82	-	-	-	-	90	96	97	0.90	*L. kirschneri*	
	L. noguchii	-	-	-	-	-	-	-	-	50	68	92	0.54	81	88	100	0.85	91	96	98	0.92	*L. noguchii*	
	L. borgpetersenii	48	65	97	0.55	-	-	-	-	-	-	-	-	-	-	-	-	90	95	99	0.92	*L. borgpetersenii*	
	L. weilii	89	96	100	0.93	88	93	100	0.905	80	94	99	0.86	-	-	-	-	72	88	96	0.77	*L. weilii*	
	L. santarosai	87	92	99	0.89	-	-	-	-	51	66	92	0.54	92	95	100	0.94	66	83	96	0.72	*L. santarosai*	
	L. alexanderi	71	87	99	0.78	-	-	-	-	81	93	99	0.86	-	-	-	-	-	-	-	-	*L. alexanderi*	
	L. alstoni	70	87	99	0.78	-	-	-	-	82	94	99	0.87	-	-	-	-	72	89	96	0.77	*L. alstoni*	
	L. kmetyi	-	-	-	-	-	-	-	-	-	-	-	-	-	-	-	-	90	94	100	0.92	*L. kmetyi*	
Intermediary	*L. wolffii*	42	67	97	0.53	-	-	-	-	96	98	99	0.96	-	-	-	-	96	98	100	0.97	*L. wolffii*	Intermediary
	L. licerasiae	100	100	100	1.00	100	100	100	1	100	100	100	1.00	100	100	100	1.00	100	100	100	1.00	*L. licerasiae*	
	L. inadai	83	92	100	0.88	-	-	-	-	90	97	100	0.94	-	-	-	-	87	94	100	0.91	*L. inadai*	
	L. fainei	-	-	-	-	-	-	-	-	89	96	100	0.93	-	-	-	-	-	-	-	-	*L. fainei*	
	L. broomii	-	-	-	-	-	-	-	-	-	-	-	-	-	-	-	-	-	-	-	-	*L. broomii*	
Saprophytic	*L. wolbachii*	78	91	100	0.85	-	-	-	-	75	89	99	0.81	-	-	-	-	26	54	93	0.37	*L. wolbachii*	Saprophytic
	L. meyeri	77	92	100	0.85	-	-	-	-	49	64	97	0.55	-	-	-	-	70	88	93	0.73	*L. meyeri*	
	L. biflexa	71	89	100	0.80	-	-	-	-	-	-	-	-	-	-	-	-	70	88	93	0.73	*L. biflexa*	
	L. vanthielii	46	68	98	0.56	-	-	-	-	52	65	92	0.54	66	83	100	0.75	27	53	93	0.37	*L. vanthielii*	
	L. terpstrae	-	-	-	-	-	-	-	-	-	-	-	-	-	-	-	-	-	-	-	-	*L. terpstrae*	
	L. yanagawae	46	69	98	0.56	-	-	-	-	49	65	97	0.55	67	83	99	0.74	-	-	-	-	*L. yanagawae*	

Conservation values (*C-value*) were expressed as a frequency between 0 and 1. The colors indicate: ■ very high conserved (0.85 ≤ *C-value* ≤ 1.0); ■ high conserved (0.7 ≤ *C-value* ≤ 0.84); ■ medium conserved (0.4 ≤ *C-value* ≤ 0.69); ■ low conserved (*C-value* ≤ 0.39); ■ no hits. Positives or cover values below 50% were discharged.

Table S5: BLAST analysis of VapCs of L. biflexa serovar Patoc 1(Ames).

		VapC of *L. biflexa* serovar Patoc strain Patoc 1 (Ames)															
***Leptospira* spp**		LBF_0418 VapC-1				LBF_2142 VapC-2				LBF_ 2175 VapC-3				LBF_2183 VapC-4			
		Identity (%)	Positives (%)	Cover (%)	*C-value*	Identity (%)	Positives (%)	Cover (%)	*C-value*	Identity (%)	Positives (%)	Cover (%)	*C-value*	Identity (%)	Positives (%)	Cover (%)	*C-value*
Pathogenic	*L. interrogans*	28	55	96	0.40	52	72	96	0.60	-	-	-	-	73	86	98	0.78
	L. kirschneri	28	55	96	0.40	-	-	-	-	-	-	-	-	74	86	98	0.78
	L. noguchii	-	-	-	-	55	73	96	0.61	-	-	-	-	72	86	98	0.77
	L. borgpetersenii	27	51	90	0.35	-	-	-	-	44	63	51	0.27	76	89	94	0.78
	L. weilii	31	52	100	0.42	51	71	96	0.59	54	74	94	0.60	72	88	97	0.78
	L. santarosai	28	53	100	0.41	55	74	96	0.62	-	-	-	-	74	86	98	0.78
	L. alexanderi	27	51	90	0.35	52	71	96	0.59	53	74	94	0.60	72	87	97	0.77
	L. alstoni	-	-	-	-	-	-	-	-	54	72	94	0.59	73	89	97	0.79
	L. kmetyi	-	-	-	-	-	-	-	-	-	-	-	-	-	-	-	-
Intermediary	*L. wolffii*	-	-	-	-	-	-	-	-	-	-	-	-	-	-	-	-
	L. licerasiae	38	52	97	0.44	-	-	-	-	-	-	-	-	65	78	79	0.56
	L. inadai	29	53	100	0.41	-	-	-	-	-	-	-	-	56	72	98	0.63
	L. fainei	-	-	-	-	-	-	-	-	-	-	-	-	-	-	-	-
	L. broomii	29	55	100	0.42	-	-	-	-	-	-	-	-	-	-	-	-
Saprophytic	*L. wolbachii*	29	51	100	0.40	91	96	100	0.94	99	99	100	0.99	81	95	100	0.88
	L. meyeri	31	54	100	0.43	93	97	100	0.95	95	97	100	0.96	93	98	100	0.96
	L. biflexa	100	100	100	1.00	100	100	100	1.00	100	100	100	1.00	100	100	100	1.00
	L. vanthielii	31	54	100	0.43	93	97	100	0.95	-	-	-	-	85	96	100	0.91
	L. terpstrae	-	-	-	-	-	-	-	-	-	-	-	-	87	94	100	0.91
	L. yanagawae	-	-	-	-	93	97	100	0.95	96	98	100	0.97	-	-	-	-

***Leptospira* spp**		LBF_2185 VapC-5				LBF_2276 VapC-6				LBF_2813 VapC-7				LBF_3285 VapC-8			
		Identity (%)	Positives (%)	Cover (%)	*C-value*	Identity (%)	Positives (%)	Cover (%)	*C-value*	Identity (%)	Positives (%)	Cover (%)	*C-value*	Identity (%)	Positives (%)	Cover (%)	*C-value*
Pathogenic	*L. interrogans*	63	83	99	0.72	76	89	100	0.83	78	88	100	0.83	51	71	96	0.59
	L. kirschneri	47	66	97	0.55	-	-	-	-	77	89	100	0.83	65	82	99	0.73
	L. noguchii	41	58	97	0.48	39	56	98	0.47	49	68	93	0.54	44	63	97	0.52
	L. borgpetersenii	48	66	97	0.55	84	93	100	0.89	-	-	-	-	65	82	99	0.73
	L. weilii	75	90	100	0.83	84	93	100	0.89	78	88	100	0.83	66	82	99	0.73
	L. santarosai	70	88	99	0.78	35	78	98	0.55	49	64	93	0.53	68	84	99	0.75
	L. alexanderi	63	83	99	0.72	83	92	100	0.88	78	90	100	0.84	65	81	99	0.72
	L. alstoni	63	83	99	0.72	77	92	100	0.85	78	88	100	0.83	65	82	99	0.73
	L. kmetyi	-	-	-	-	-	-	-	-	-	-	-	-	-	-	-	-
Intermediary	*L. wolffii*	43	65	97	0.52	37	54	96	0.44	75	88	98	0.80	65	86	99	0.75
	L. licerasiae	71	89	100	0.80	72	87	99	0.79	77	90	98	0.82	47	68	96	0.55
	L. inadai	70	87	100	0.79	33	55	74	0.33	75	88	98	0.80	42	62	96	0.50
	L. fainei	-	-	-	-	-	-	-	-	73	88	98	0.79	-	-	-	-
	L. broomii	-	-	-	-	-	-	-	-	-	-	-	-	-	-	-	-
Saprophytic	*L. wolbachii*	89	95	100	0.92	36	57	97	0.45	83	91	100	0.87	87	97	100	0.92
	L. meyeri	77	90	100	0.84	95	98	100	0.97	48	63	93	0.52	84	97	100	0.91
	L. biflexa	100	100	100	1.00	100	100	100	1.00	100	100	100	1.00	100	100	100	1.00
	L. vanthielii	44	68	100	0.56	95	98	100	0.97	50	65	93	0.53	88	97	100	0.93
	L. terpstrae	-	-	-	-	-	-	-	-	-	-	-	-	-	-	-	-
	L. yanagawae	47	70	97	0.57	37	53	97	0.44	50	65	93	0.53	92	97	100	0.95

Conservation values (C-value) were expressed as a frequency between 0 and 1. The colors indicate: ■ very high conserved (0.85 ≤ C-value ≤ 1.0); ■ high conserved (0.7 ≤ C-value ≤ 0.84); ■ medium conserved (0.4 ≤ C-value ≤ 0.69); ■ low conserved (C-value ≤ 0.39); ■ no hits. Positives or cover values below 50% were discharged.

Table S6: Conservation of the VapCs of *L. interrogans* serovar Copenhageni strain Fiocruz L1-130 with some human pathogenic bacteria

Pathogenic Bacteria	VapC of *L. interrogans* serovar Copenhageni str. Fiocruz L1-130															
	LIC10866 VapC-1				LIC12116 VapC-2				LIC12660 VapC-3				LIC12713 VapC-4			
	Identity (%)	Positives (%)	Cover (%)	*C-value*	Identity (%)	Positives (%)	Cover (%)	*C-value*	Identity (%)	Positives (%)	Cover (%)	*C-value*	Identity (%)	Positives (%)	Cover (%)	*C-value*
Escherichia coli	-	-	-	-	-	-	-	-	40	65	98	0.51	29	56	94	0.40
Klebsiella pneumoniae	-	-	-	-	-	-	-	-	40	60	98	0.49	-	-	-	-
Mycobacterium tuberculosis	-	-	-	-	41	55	96	0.46	-	-	-	-	-	-	-	-
Neisseria gonorrhoeae	-	-	-	-	-	-	-	-	-	-	-	-	-	-	-	-
Neisseria meningitidis	-	-	-	-	-	-	-	-	-	-	-	-	38	53	92	0.42
Proteus mirabilis	-	-	-	-	-	-	-	-	45	63	98	0.53	-	-	-	-
Pseudomonas aeruginosa	36	56	93	0.43	-	-	-	-	38	62	98	0.49	-	-	-	-
Serratia marcescens	-	-	-	-	-	-	-	-	42	60	98	0.50	40	54	92	0.43
Shigella sonnei	-	-	-	-	-	-	-	-	38	63	98	0.49	-	-	-	-
Staphylococcus aureus	-	-	-	-	-	-	-	-	34	55	99	0.44	-	-	-	-
Streptococcus pneumoniae	-	-	-	-	39	55	60	0.28	42	65	98	0.52	-	-	-	-
Vibrio cholerae	-	-	-	-	-	-	-	-	34	62	97	0.47	-	-	-	-

Conservation values (C-value) were expressed as a frequency between 0 and 1. The colors indicate: medium conserved (0.4 ≤ C-value ≤ 0.69); low conserved (C-value ≤ 0.39); no hits. Positives or cover values below 50% were discharged.
